# Supplementary material for: Perception of root‐active CLE peptides requires CORYNE function in the phloem vasculature
Source: EMBO Rep. 2017 Jun 12;18(8):1367–81. doi: 10.15252/embr.201643535 (PMC5538625; doi:10.15252/embr.201643535)
Supplement: Supplementary file 1 — Expanded View Figures PDF [file EMBR-18-0-s001.pdf]

Expanded View Figures

Figure EV1. BAM3-CLE45 control experiments and *bam3* alleles.

A Relative primary root length of indicated genotypes at 9 dag, in response to increasing amounts of CLE45 in the media. n = 12 for each genotype, mean ± s.e.m. All differences as compared to wild type were statistically significant (Student's t-test) with $P < 0.001$ for 15 and 50 nM, and $P < 0.05$ for 100 nM.

B ITC of purified PXY extracellular domain vs. CLV41/44 peptide. n.d.: not detectable. N: stoichiometry, K_d dissociation constant. Shown are experimental values ± fitting errors (95% confidence interval).

C ITC of purified BAM3 extracellular domain vs. CLV3 peptide.

D ITC of purified BAM3 extracellular domain vs. an N-terminally tyrosine-modified CLV3 peptide.

E ITC of purified BAM3 extracellular domain vs. an N-terminally tyrosine-modified CLE45 peptide.

F Representative 9-day-old Col-0 seedlings grown on mock or in presence of indicated peptides.

 © 2017 The Authors

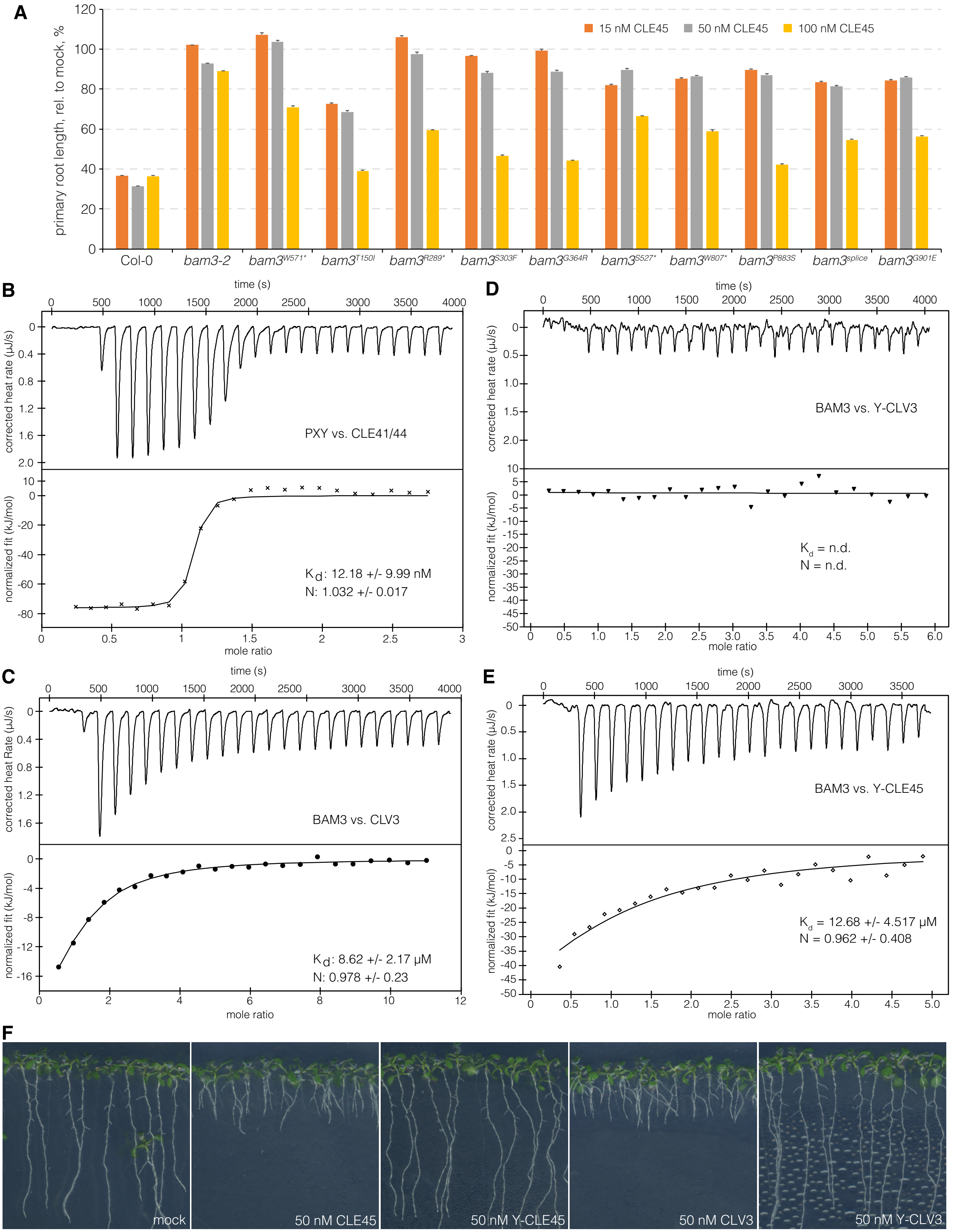
A
primary root length, rel. to mock, %
15 nM CLE45
50 nM CLE45
100 nM CLE45
Col-0
bam3-2
bam3W571*
bam3T150I
bam3R289*
bam3S303F
bam3G364R
bam3S527*
bam3W807*
bam3P883S
bam3splice
bam3G901E
B
time (s)
corrected heat rate (μJ/s)
PXY vs. CLE41/44
normalized fit (kJ/mol)
Kd: 12.18 +/- 9.99 nM
N: 1.032 +/- 0.017
mole ratio
D
time (s)
corrected heat rate (μJ/s)
BAM3 vs. Y-CLV3
normalized fit (kJ/mol)
Kd = n.d.
N = n.d.
mole ratio
C
time (s)
corrected heat Rate (μJ/s)
BAM3 vs. CLV3
normalized fit (kJ/mol)
Kd: 8.62 +/- 2.17 μM
N: 0.978 +/- 0.23
mole ratio
E
time (s)
corrected heat rate (μJ/s)
BAM3 vs. Y-CLE45
normalized fit (kJ/mol)
Kd = 12.68 +/- 4.517 μM
N = 0.962 +/- 0.408
mole ratio
F
mock
50 nM CLE45
50 nM Y-CLE45
50 nM CLV3
50 nM Y-CLV3

Figure EV1.

Figure EV2. BAM3 localization and biochemical control experiments.

- A Transient expression of BAM3 wild-type or mutant $BAM3^{QYY}$ CITRINE fusion proteins (green fluorescence) in tobacco leaf epidermal cells, under control of a constitutive promoter (confocal microscopy).
- B Close-up of developing protophloem sieve element cell files expressing BAM3 wild-type or mutant $BAM3^{QYY}$ CITRINE fusion proteins (green fluorescence).
- C, D Primary root length of 7-day-old seedlings of indicated genotypes on mock or CLE45 media, several independent lines per transgene construct are shown. Differences as compared to mock are not statistically significant unless indicated (Student's *t*-test); *$P < 0.05$; **$P < 0.01$; ***$P < 0.001$; mean ± s.e.m.
- E Transphosphorylation kinase assays with purified BAM3 kinase domain (BAM3-KD) or SERK1 kinase domain (SERK1-KD) as well as kinase dead point mutant versions (mBAM3-KD & mSERK1-KD) alone and in combination.
- F Analytical size-exclusion chromatography of purified BAM3 and SERK3 extracellular domains in the presence of CLE45 peptide reveals no ligand-induced complex formation between BAM3 and SERK3.
- G Analytical size-exclusion chromatography of purified PXY and SERK1 extracellular domains in the presence of CLE41/44 peptide reveals CLE41/44-induced binding of SERK1 to the PXY ectodomain.
- H Expression of SERK1-CITRINE fusion protein (green fluorescence) under control of the native *SERK1* promoter (blue fluorescence: calcofluor white cell wall staining). Green channel is shown separately (left) and in overlay with blue channel (right). Asterisk indicates the developing sieve element cell file.

© 2017 The Authors

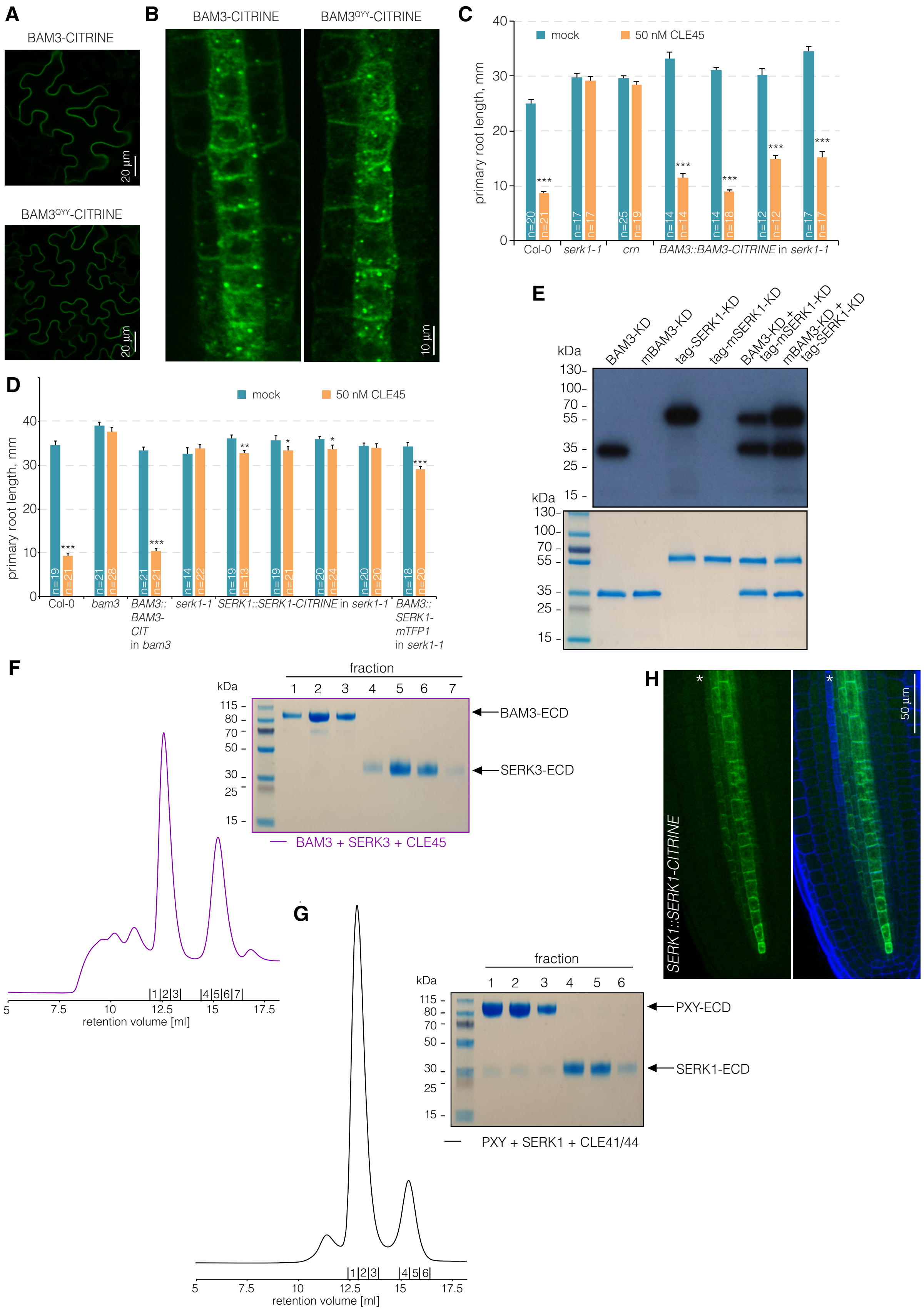

A
BAM3-CITRINE
20 µm
BAM3QYY-CITRINE
20 µm
B
BAM3-CITRINE
BAM3QYY-CITRINE
10 µm
C
mock
50 nM CLE45
primary root length, mm
***
Col-0
serk1-1
crn
BAM3::BAM3-CITRINE in serk1-1
D
mock
50 nM CLE45
primary root length, mm
Col-0
bam3
BAM3::BAM3-CIT in bam3
serk1-1
SERK1::SERK1-CITRINE in serk1-1
BAM3::SERK1-mTFP1 in serk1-1
E
kDa
BAM3-KD
mBAM3-KD
tag-SERK1-KD
tag-mSERK1-KD
BAM3-KD + tag-mSERK1-KD
mBAM3-KD + tag-SERK1-KD
F
fraction
BAM3-ECD
SERK3-ECD
BAM3 + SERK3 + CLE45
retention volume [ml]
G
fraction
PXY-ECD
SERK1-ECD
PXY + SERK1 + CLE41/44
retention volume [ml]
H
SERK1::SERK1-CITRINE
50 µm

Figure EV2.

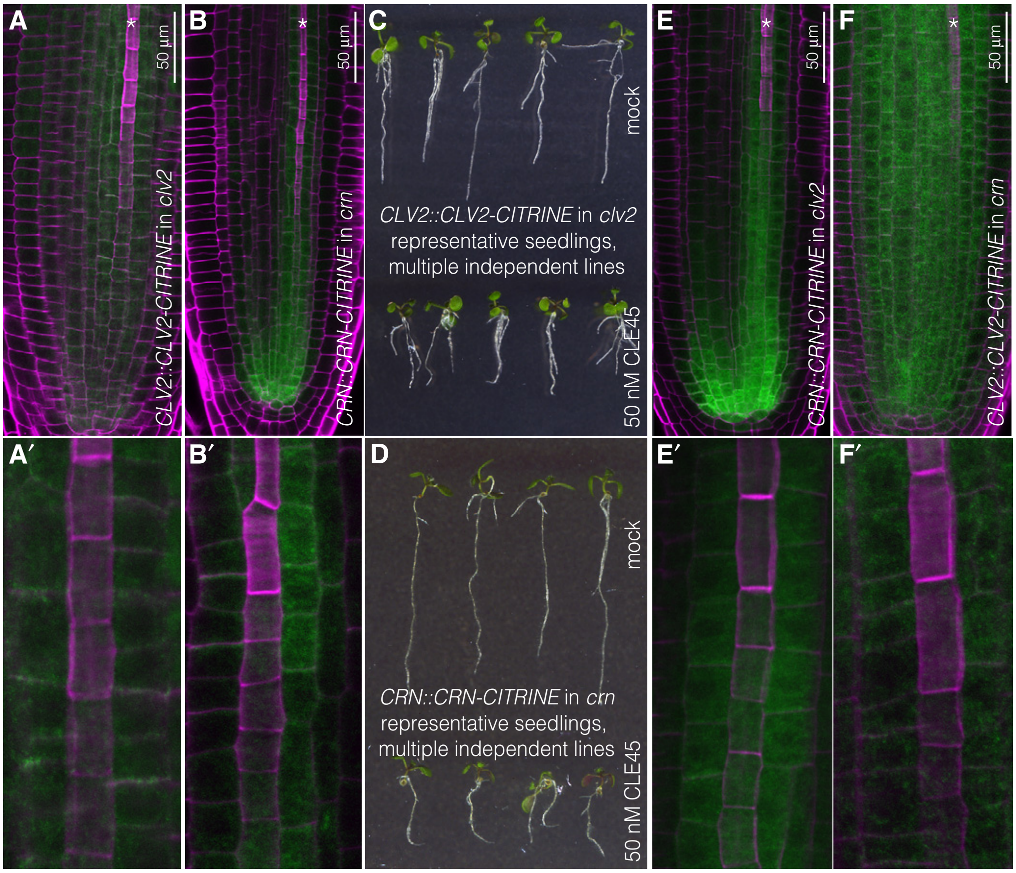

Figure EV3. CLV2 and CRN localizations in *Arabidopsis* roots.

A Expression of CLV2-CITRINE fusion protein (green fluorescence) under control of the native promoter in *clv2* root meristems (magenta fluorescence: calcofluor white cell wall staining) (confocal microscopy). Asterisk marks developing protophloem sieve element strand. Close-up (A′) with developing protophloem at the center is shown.

B Same as in (A), for CRN-CITRINE fusion protein under control of the native promoter in *crn* root meristems.

C Representative 5-day-old *clv2* seedlings expressing CLV2-CITRINE fusion protein under control of its native promoter grown on mock or CLE45.

D Representative 5-day-old *crn* seedlings expressing CRN-CITRINE fusion protein under control of its native promoter grown on mock or CLE45.

E Same as in (A), for CRN-CITRINE fusion protein under control of the native promoter in *clv2* root meristems.

F Same as in (A), for CLV2-CITRINE fusion protein under control of the native promoter in *crn* root meristems.

 © 2017 The Authors

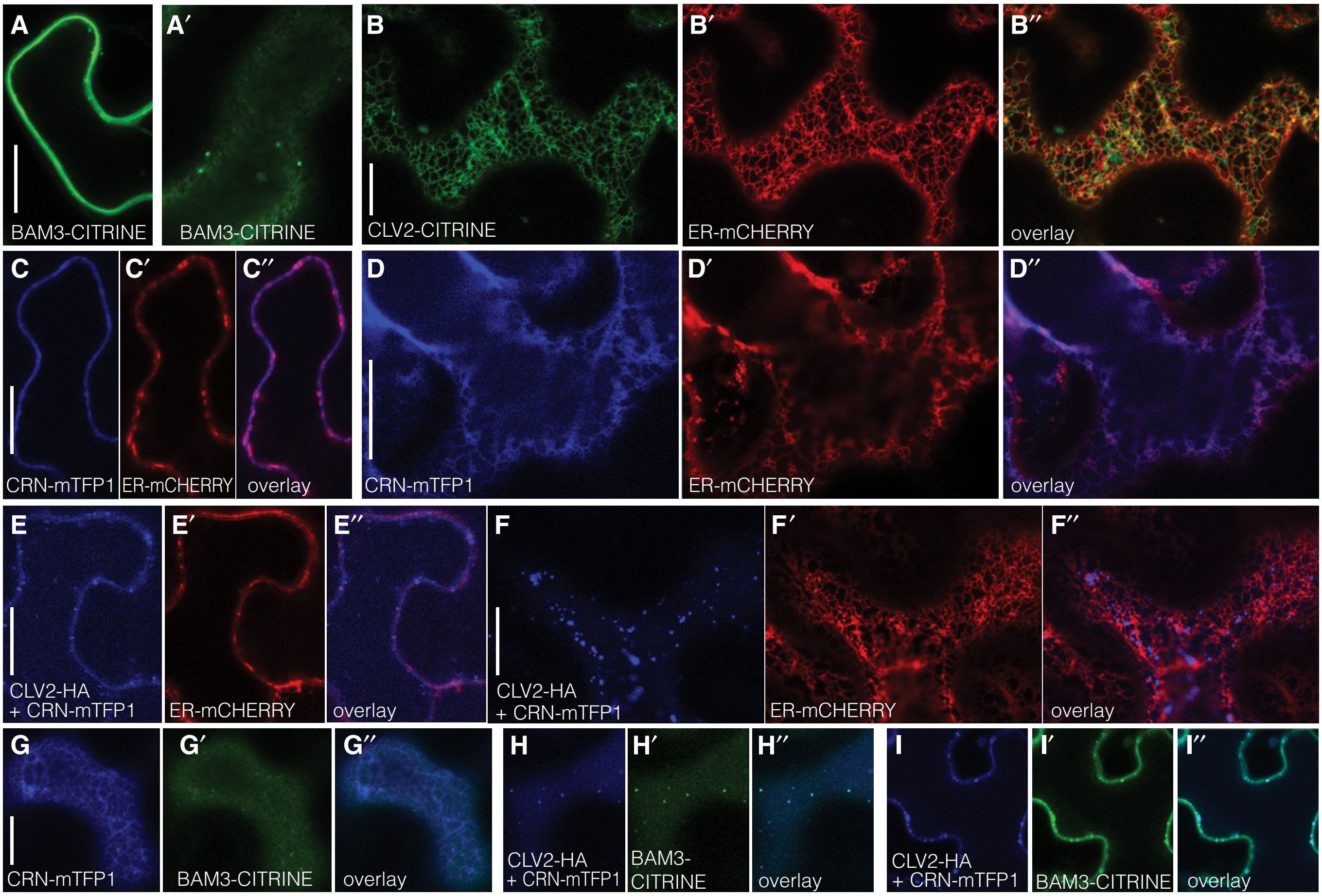

Figure EV4. Tobacco co-localization, additional, and control experiments.

A Transient expression of BAM3-CITRINE fusion protein (green fluorescence) in tobacco (*Nicotiana benthamiana*) leaf epidermal cells, under control of a constitutive promoter (confocal microscopy), optical section through cell center. Panel (A′): same in cell surface view.

B Transient co-expression of CLV2-CITRINE fusion protein (green fluorescence) and an endoplasmic reticulum marker (ER-mCHERRY, red fluorescence).

C Transient co-expression of CRN-mTFP1 fusion protein (blue fluorescence) and the ER-mCHERRY marker (red fluorescence), optical section through cell center.

D Same as (C), in cell surface view.

E, F Corresponding to (C) and (D), in the additional presence of (non-fluorescent) CLV2-HA fusion protein.

G Transient co-expression of CRN-mTFP1 (blue fluorescence) and BAM3-CITRINE (green fluorescence) fusion proteins, in cell surface view.

H, I Transient co-expression of CRN-mTFP1 (blue fluorescence) and BAM3-CITRINE (green fluorescence) fusion proteins, in the additional presence of (non-fluorescent) CLV2-HA fusion protein. Panel (H): cell surface view. Panel (I): optical section through cell center.

Data information: Scale bars are 20 μm.

© 2017 The Authors

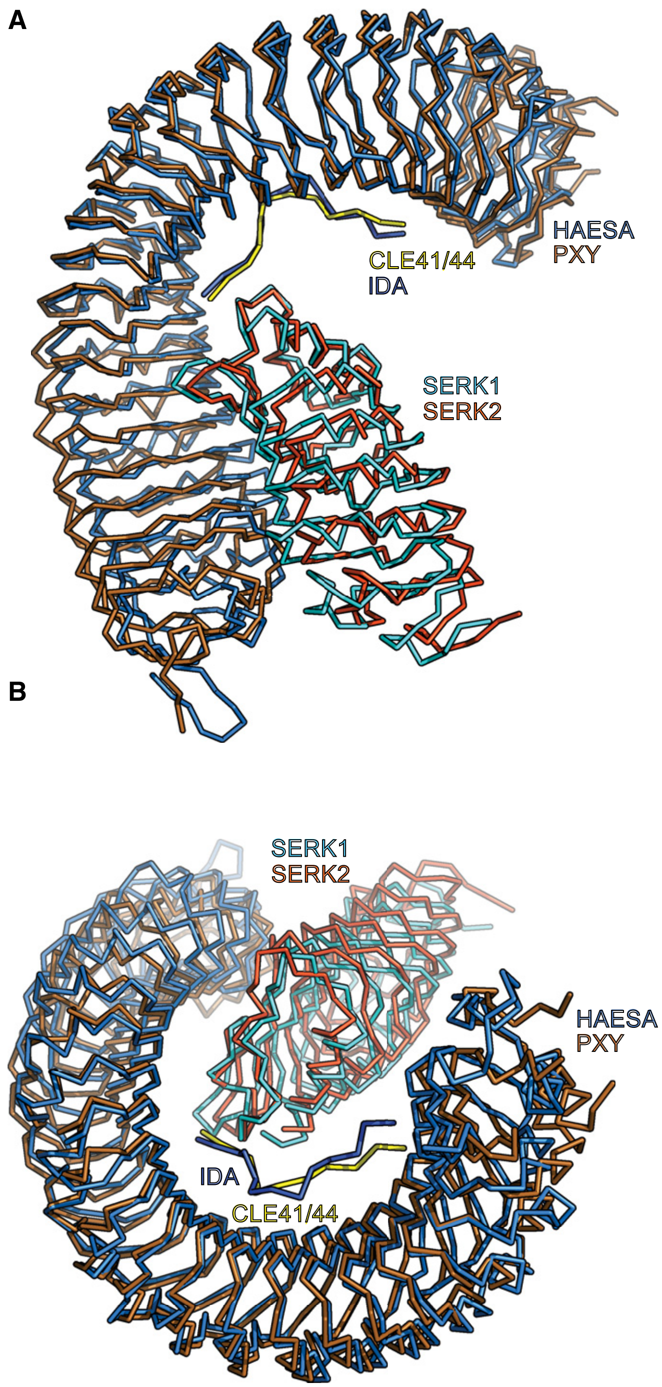

Figure EV5. Structure comparison of HAESA-IDA-SERK1 and PXY-CLE41-SERK2 signaling complexes.

A, B Structural superposition of HAESA-IDA-SERK1 (PDB-ID 5IYX, HAESA in light blue, IDA in dark blue, and SERK1 in cyan) (Santiago *et al*[30]) with a PXY-CLE41/44-SERK2 complex (PDB-ID 5GQR, PXY in gold, IDA in yellow, and SERK2 in orange) (Zhang *et al*[45]). The complexes closely align with an r.m.s.d. of 2.3 Å comparing 770 C_α atoms.

 © 2017 The Authors
