# Supplementary material for: Perception of root‐active CLE peptides requires CORYNE function in the phloem vasculature
Source: EMBO Rep. 2017 Jun 12;18(8):1367–81. doi: 10.15252/embr.201643535 (PMC5538625; doi:10.15252/embr.201643535)
Supplement: Supplementary file 2 — Table EV1 [file EMBR-18-0-s002.docx]

Table EV1. Transgenic and mutant lines created in this study

| **Number** | **Name** | **Method** |
| --- | --- | --- |
| 1 | *BAM3::BAM3-CITRINE* in *wt*, *bam3-2*, *brx-2 bam3*, *serk1-1* and *crn-10* | transformation |
| 2 | *BAM3::bam3^QYY^-CITRINE* in *bam3-2*, *brx-2 bam3* | transformation |
| 3 | *BAM3::CRN-CITRINE* in *wt*, *crn-10*, *clv2-1* | transformation |
| 4 | *BAM3::SERK1-mTFP1* in *serk1-1* | transformation |
| 5 | *SERK1::SERK1-CITRINE in wt and serk1-1* | transformation |
| 6 | *CRN::CRNg-CITRINE in crn-10 and clv2-1* | transformation |
| 7 | *CLV2::CLV2g-CITRINE in clv2-1 and crn-10* | transformation |
| 8 | *MAKR5::MAKR5-GFP in crn-10 and clv2-1* | transformation |
| 9 | *crn-10 brx-2* | cross |
| 10 | *serk1-1 brx-2* | cross |
| 11 | *clv2-1 brx-2* | cross |
