# Supplementary material for: Perception of root‐active CLE peptides requires CORYNE function in the phloem vasculature
Source: EMBO Rep. 2017 Jun 12;18(8):1367–81. doi: 10.15252/embr.201643535 (PMC5538625; doi:10.15252/embr.201643535)
Supplement: Supplementary file 3 — Table EV2 [file EMBR-18-0-s003.docx]

Table EV2. Constructs used in this study

| **Number** | **Name** | **Source** |
| --- | --- | --- |
| IH_p42 | *pH7-m34GW-35S::BAM3-YN* | This study |
| IH_p43 | *pH7-m34GW-35S::BAM3-YC* | This study |
| IH_p48 | *pH7-m34GW-35S::CLV2-YN* | This study |
| IH_p49 | *pH7-m34GW-35S::CLV2-YC* | This study |
| IH_p50 | *pH7-m34GW-35S::CRN-YN* | This study |
| IH_p51 | *pH7-m34GW-35S::CRN-YC* | This study |
| IH_p52 | *pH7-m34GW-35S::BRI1-YN* | This study |
| IH_p53 | *pH7-m34GW-pBAM3::BAM3-CITRINE* | Rodriguez-Villalon et al, 2014 |
| IH_p54 | *pH7-m34GW-pBAM3::bam3^QYY^-CITRINE* | This study |
| IH_p55 | *pH7-m34GW-pSERK1::SERK1-CITRINE* | This study |
| IH_p56 | *pH7-m34GW-pMAKR5::MAKR5-GFP* | Kang and Hardtke, 2016 |
| IH_p57 | *pH7-m34GW-pBAM3::CRN-CITRINE* | This study |
| IH_p58 | *pH7-m24GW-pCLV2CLV2g-CITRINE* | This study |
| IH_p59 | *pH7-m24GW-pCRNCRNg-CITRINE* | This study |
| IH_p60 | *pH7-m34GW-pUBQ10::BAM3-CITRINE* | This study |
| IH_p61 | *pH7-m34GW-35S::BAM3-mTFP1* | This study |
| IH_p62 | *pH7-m34GW-35S::BRI1-mTFP1* | This study |
| IH_p63 | *pH7-m34GW-35S::CLV2-HA* | This study |
| IH_p64 | *pH7-m34GW-35S::SERK1-TurboRFP* | This study |
| IH_p65 | *pH7-m34GW-35S::CRN-TurboRFP* | This study |
| IH_p66 | *pH7-m34GW-35S::SERK1-TurboRFP* | This study |
| IH_p67 | *pH7-m34GW-35S::CLV2-CITRINE* | This study |
| IH_p68 | *pH7-m34GW-pBAM3::SERK1-mTFP1* | This study |
| IH_69 | *pH7-m34GW-35S::CRN-mTFP1* | This study |
| IH_70 | *pH7-m34GW-35S::BRI1-YC* | This study |
| IH_71 | *pH7-m34GW-35S::BKI1-YN* | This study |
| p1 | *pFastBac1-Az-BAM3-ECD (30-651)* | This study |
| p2 | *pFastBac1-Az-PXY-ECD (30-647)* | This study |
| p3 | *pFastBac1-Az-SERK1-ECD (24-213)* | This study |
| p4 | *pFastBac1-Az-SERK3-ECD (22-220)* | This study |
| p5 | *pET-TH-BAM3-KD (679-992)* | This study |
| p6 | *pET-TH-SERK1 (264–625)* | This study |
