# Supplementary material for: Perception of root‐active CLE peptides requires CORYNE function in the phloem vasculature
Source: EMBO Rep. 2017 Jun 12;18(8):1367–81. doi: 10.15252/embr.201643535 (PMC5538625; doi:10.15252/embr.201643535)
Supplement: Supplementary file 4 — Table EV3 [file EMBR-18-0-s004.docx]

Table EV3. Primers used in this study

| **Number** | **Name** | **Sequence** | **Comments** |
| --- | --- | --- | --- |
| 1 | CLV2attb1F1 | GTA CAA AAA AGC AGG CTC CAT GAT AAA GAT TGC AGA TTT CAC TC | Cloning CLV2 cds into pENTRY221 |
| 2 | CLV2attb1F2 | GGG GAC AAG TTT GTA CAA AAA AGC AGG CTC CAT GAT AAA GA | Cloning CLV2 cds into pENTRY221 |
| 3 | CLV2attb2R1 | TTG TAC AAG AAA GCT GGG TAA  GCT TTG GTC TGA AGA ATA TAA CTA C | Cloning CLV2 cds into pENTRY221 |
| 4 | CLV2attb2R2 | GGG GAC CAC TTT GTA CAA GAA AGC TGG GTA AGC TTT GGT CTG A | Cloning CLV2 cds into pENTRY221 |
| 5 | CRNattb1F1 | GTA CAA AAA AGC AGG CTC CAT GAA GCA AAG AAG AAG AAG AAA | Cloning CRN cds into pENTRY221 |
| 6 | CRNattb1F2 | GGG GAC AAG TTT GTA CAA AAA AGC AGG CTC CAT GAA GCA AAG A | Cloning CRN cds into pENTRY221 |
| 7 | CRNattb2R1 | TTG TAC AAG AAA GCT GGG TAA AAG CTG TGC AGT TGT GTA GCA | Cloning CRN cds into pENTRY221 |
| 8 | CRNattb2R2 | GGG GAC CAC TTT GTA CAA GAA AGC TGG GTA AAA GCT GTG CAG | Cloning CRN cds into pENTRY221 |
| 9 | pCLV2CLV2g_ attb4F | GGG GAC AAC TTT GTA TAG AAA AGT TGC ATA TTA GAT CTA GGG TTT AGA TAC CAT T | Cloning full genomic pCLV2-CLV2 |
| 10 | pCLV2CLV2g_ attb1R | GGG GAC TGC TTT TTT GTA CAA ACT TGG AGC TTT GGT CTG AAG AAT ATA AC | Cloning full genomic pCLV2-CLV2 |
| 11 | pCRNCRNg_attb4F | GGG GAC AAC TTT GTA TAG AAA AGT TGC AAT TTT GGT TTT GAA TCT GTG TC | Cloning full genomic pCRN-CRN |
| 12 | pCRNCRNg_attb1R | GGG GAC TGC TTT TTT GTA CAA ACT TGG AAA GCT GTG CAG TTG TGT AAG | Cloning full genomic pCRN-CRN |
| 13 | pSERK1 KpnI F | TGG TAC CCG TTT CTC TTT CAT AAC AAG GTA GC | Cloning SERK1 promoter |
| 14 | pSERK1 SmaI R | TCC CGG GTT CAA ACA ACA ATG CTA AAT TTC G | Cloning SERK1 promoter |
| 15 | crn F | GTA GAA GCA GCA ATG AAG CAA AGA AGA AGGTG | Genotyping of *crn* mutant (PCR product was later cut with HphI) |
| 16 | crn R | GTT GAA GTT GTG GAT AAG TG | Genotyping of *crn* mutant |
